# 宣威地区所产烟煤燃烧产出可吸入细颗粒物与肺癌发病的关系研究

**DOI:** 10.3779/j.issn.1009-3419.2015.07.03

**Published:** 2015-07-20

**Authors:** 加鹏 杨, 宇 曹, 云超 黄, 光剑 李, 联华 叶, 光强 赵, 玉洁 雷, 小波 陈, 林玮 田

**Affiliations:** 1 650118 昆明，昆明医科大学第三附属医院/云南省肿瘤医院胸外一科 Department of Thoracic Surgery 1 Ward, the Third Affiliated Hospital of Kunming Medical University/Yunnan Provincial Tumor Hospital, 650118 Kunming, China; 2 650000 昆明，昆明医科大学附属延安医院/昆明市延安医院心外科 Department of Cardiac Surgery, the Yan'an Affiliated Hospital of Kunming Medical University/Yan'an Hospital of Kunming City, 650000 Kunming, China; 3 999077 香港，香港大学公共卫生学院 School of Public Health, University of Hong Kong, 999077 Hong Kong, China

**Keywords:** 肺肿瘤, 宣威, 可吸入细颗粒物, Lung neoplasms, Xuanwei, Fine particulate matter

## Abstract

**背景与目的:**

云南省宣威地区是中国乃至世界肺癌的高发区，肺癌已成为制约当地社会经济发展和影响社会民生的重要因素。煤炭是当地主要的生活燃料，燃煤是当地室内污染的主要来源。本研究探讨云南宣威不同肺癌发病率地区烟煤燃烧过程中可吸入细颗粒物(fine particulate matter, PM2.5)产出情况，以及不同地区PM2.5成分异同。探讨吸入细颗粒物与当地肺癌高发的关系。

**方法:**

收集宣威市来宾镇老林煤矿C1煤层、宝山镇虎场煤矿K7煤层、文兴镇太平煤矿M30煤层的煤矿进行燃烧试验。收集室内的空气中的PM2.5进行称重，元素分析，用电子显微镜观察其形态，对比三种PM2.5异同。对宣威地区的肺癌患者的术后标本进行电子显微镜观察。

**结果:**

室内空气中的PM2.5浓度分别为C1煤(8.244±1.460)mg/m^3^，K7煤(5.066±0.984)mg/m^3^，K7煤(5.071±1.460)mg/m^3^；三组空气中PM2.5浓度两两比较差异有统计学意义(*Ρ*=0.029)。C1煤层中滤膜上的杂质有(Silicon, Si)和氧(Oxygen, O)元素富集，三组滤膜上均发现了碳(Carbon, C)，硫(Sulfur, S)的聚集，在部分的滤膜上可见游离的二氧化硅(SiO_2_)，部分滤膜上有铝(Aluminium, Al)、钙(Calcium, Ca)元素的聚集。C1煤层与其他煤层相比所产生颗粒物形态不规则，成团块状，杂质较多。在部分的宣威来宾地区的肺癌患者术后标本中，发现纳米级细颗粒的杂质。

**结论:**

C1煤与K7和M30煤燃烧产生的PM2.5不同，PM2.5的成分可能与当地肺癌高发相关。

## Abstract (en)

**Background and objective:**

The high incidence of lung cancer in Xuanwei, China, has become an important restricting factor for livelihood development, thus exerting local social and economic impacts. Coal is the main fuel of the local community and also the main source of indoor pollution. This study aims to explore the coal combustion inhalable fine particulate matter (PM2.5) and its component output differences in different areas of Xuanwei, Yunnan. Moreover, the aim of this study is to investigate the relationship between inhalation of fine particles and high incidence of local lung cancer.

**Methods:**

For combustion test, coal mines designated as C1, K7 and M30 were collected from LaoLin Colliery of Laibing Town, Huchang Colliery of Baoshan Town, and Taiping Colliery of Wenxing Town in Xuanwei, respectively. PM2.5 of indoor air was weighed, analyzed for elemental composition, and morphologically compared. The pathological specimen of lung cancer patients in Xuanwei who underwent operation was observed through electron microscope.

**Results:**

The PM2.5 concentrations in indoor air were (8.244 ±1.460) mg/m^3^ (C1), (5.066±0.984) mg/m^3^ (K7), and (5.071±1.460) mg/m^3^ (M30). The differences among pairwise comparisons were statistically significant (P=0.029). The filter impurities of C1 coal seam primarily include Si-and O-enriched compounds. Moreover, three membranes that comprised other elements, including C, S, and Si, were observed. These membranes were evident from the aggregation of silica and a Ca-Al membrane. Compared with that of other coal seams, C1 coal generated a mass of impurities, in which several particles have irregular shape. We found nanoscale fine particles in some specimens of Xuanwei lung cancer patients.

**Conclusion:**

The produced combustion of C1 coal was different from that of K7 and M30 coal. PM2.5 composition may be associated with the high local incidence of lung cancer.

云南省宣威地区是中国乃至世界肺癌的高发区，据2005年当地流行病学资料显示，肺癌发病率来宾镇为128.31/10万^[1, 2]^。肺癌已成为制约当地社会经济发展和影响社会民生的重要因素。有研究^[3-5]^表明，其原因可能与所烧燃煤相关。宣威地区地处云南东北部，拥有丰富的煤炭资源，该区煤层属于滇东黔西晚二叠纪聚煤区，该煤区C1煤层中纳米二氧化硅的富集^[3]^。在宣威各乡镇的肺癌发病率也不尽相同(宝山镇18/10万，文兴镇5.83/10万)，呈点状高发。寻找该地区肺癌的发病原因以及发病机制成为研究的热点和难点。然而煤炭是当地主要的生活燃料，燃煤是当地室内污染的主要来源。一些研究^[4, 5]^表明宣威肺癌高发与室内烟煤燃烧产生的多环芳烃等污染物以及纳米级二氧化硅有关。

本研究根据当地流行病资料，取不同肺癌发病率地区所产煤样，进行燃烧试验，对可吸入细颗粒物(PM2.5)成分进行分析，探讨可吸入细颗粒物特征；了解细颗粒物在宣威地区的肺癌患者的术后标本中的赋存情况。

## 1 材料与方法

### 1.1 材料

分别选取宣威市来宾镇老林煤矿C1煤层、宝山镇虎场煤矿K7煤层、文兴镇太平煤矿采集M30煤层、原煤各30份，每份2 kg(由云南省煤炭研究院提供)；过氯乙烯尘测滤膜，购自北京康农兴牧科技发展中心公司；病理组织由昆明医科大学第三附属医院病理科提供。

### 1.2 仪器和设备

Sartorius电子分析天平德国Sartorius公司；XL30ESEM-TMP扫描电子显微镜荷兰飞利浦公司；Phoenix+DIM一体化能谱及电子背散射衍射仪美国EDAXH800透射电子显微镜日本日立公司；ZSX100e型波长色散X射线荧光光谱仪日本理学(Rigaku)公司；TTR-Ø型转靶多功能X射线衍射仪日本理学(Rigaku)公司；空气污染检测仪(PM2.5切割器、采样系统)TE-6070VFC/Tisch Environmental Equipment. Ltd, USA。

### 1.3 烟煤燃烧方法

将所取煤样切割成约5 cm×5 cm×5 cm的方形小块，置于开放式火塘中，用液化气喷灯引燃，每个煤样燃烧时间约为30 min，同时采用空气污染检测仪对室内空气进行恒流采样(30 min, 100 L/min)。

### 1.4 空气采样方法

将校正好的空气细颗粒物采样仪器放置在模拟燃烧室内(3.5 m×4 m×5 m)，固定在三角架上，使采样头(PM2.5切割器、采样系统：切割粒径Da50=(2.5±0.2)μm；单次采样结束后更换滤膜，各煤样分别采样滤膜30张。将完好的滤膜分别放入单独的封口袋中，密封并置于4 oC冰箱中保存。采样前后对滤膜进行称重备用。重量法测定空气中可吸入细颗粒物的浓度。

### 1.5 空气滤膜检测方法

采用X射线荧光光谱仪(X-ray fluorescence spectrometry, XRF)对滤膜进行分析F(9)-U(92)之间所有元素的检测分析。采用X射线衍射仪(X-ray diffraction, XRD)对滤膜上晶体结构测定分析功能的检测分析。采用扫描电子显微镜SEM对滤膜上的颗粒物形态进行观察。透射电子显微镜对滤膜进行观察，同时行能量分散X射线(EDX)分析检测。透射电子显微镜对滤膜进行观察宣威地区的肺癌组织标本，寻找可吸入颗粒物，并对颗粒物进行定位。

### 1.6 统计学方法

采用SPSS 17.0统计软件分析，数据结果以Mean±SD的方式显示，多组间均数比较采用单因素方差分析(*ANOVA*)，*P* < 0.05为差异具有统计学意义。

## 2 结果

### 2.1 各组烟煤燃烧产生的PM2.5

三组烟煤燃烧后经重量法计算空气中每30 min的PM2.5浓度分别为C1煤(8.244±1.460)mg/m^3^、K7煤(5.066±0.984)mg/m^3^、M30煤(5.071±0.869)mg/m3；将C1组与K7、M30组经过比较空气中PM2.5浓度差异有统计学意义(*Ρ*=0.029)。

### 2.2 各组PM2.5的X射线荧光光谱仪结果

通过X射线荧光光谱仪(XRF)对三种不同的滤膜进行检测。发现C1煤层燃烧产物中滤膜上的杂质有Si和O元素富集，其他两个地方的较少(图 1)，此外，三组滤膜上否发现了C、S、O的聚集，部分滤膜上有Ca、Al元素的聚集(图 1)。

**1:**
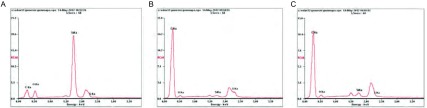
XRF结果。A：C1煤燃烧产物的XRF；B：K7煤燃烧产物的XRF；C：M30煤燃烧产物的XRF。 XRF results. A: Result of the C1 Coal combustion products XRF; B: Result of the K7 Coal combustion products XRF; C: Result of the M30 Coal combustion products XRF. XRF: X-ray fluorescence spectrometry.

### 2.3 各组PM2.5的X射线衍射结果

通过XRD对三种不同的滤膜进行检测发现C1煤燃烧烟尘中含有结晶型SiO_2_细颗粒物，而K7和M30煤燃烧烟尘中未检测到结晶型SiO_2_细颗粒物存在(图 2)。

**2:**
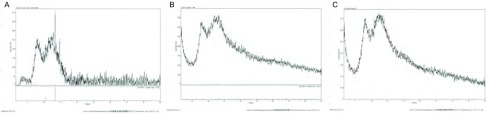
XRD结果。A：C1煤层XRD检测结果；B：K7煤层XRD检测结果；C：M30煤层XRD检测结果。 XRD results. A: C1 coal XRD test results; B: K7 coal XRD test results; C: M30 coal XRD test results.

### 2.4 三组滤膜上颗粒物的形态各不相同

扫描电镜观察三种烟煤燃烧的PM2.5的情况，C1煤层的燃烧产物与K7和M30煤层相比所产生的颗粒物形态不规则，成团块状，含有较多的杂质(图 3，图 4)。透射电镜下观察C1煤燃烧后烟尘中PM2.5形态及能谱分析，通过透射电镜观察C1煤烟尘中分离出的含硅颗粒物形态，发现这些颗粒物表面不光滑，形态欠规整，取其中颗粒物测量直径为30.74 nm；用透射电镜装配的能谱仪分析，此颗粒物中含有Si、Ca、O等元素，其中以Si元素含量最高(图 5)。

**3:**
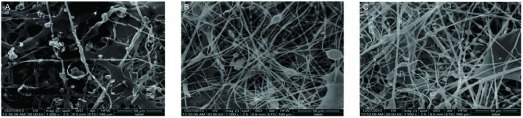
可吸入颗粒物图片。A：过氯乙烯滤膜上截留的C1煤燃可吸入颗粒物(×1, 500)；B：过氯乙烯滤膜上截留的K7煤燃可吸入颗粒物(×1, 500)；C：过氯乙烯滤膜上截留的M30煤燃可吸入颗粒物(×1, 500)。 Respirable particulate matter pictures. A: Ethylene perchloride membrane on intercept C1 coal burning particulate matter (×1, 500); B: Ethylene perchloride membrane on intercept K7 coal burning particulate matter (×1, 500); C: Ethylene perchloride membrane on intercept M30 coal burning particulate matter (×1, 500).

**4:**
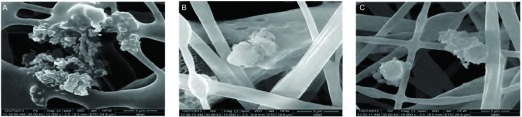
颗粒物局貌。A：C1颗粒物局貌(×10, 000)；B：K7颗粒物局貌(×10, 000)；C：M30颗粒物局貌(×10, 000)。 Particle board looks. A: C1 particle board looks; B: K7 particle board looks; C: M30 particle board looks.

**5:**
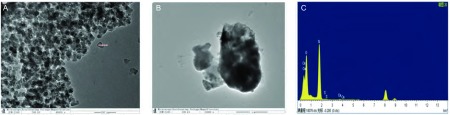
二氧化硅颗粒物。A：经分离提纯后滤膜上(C1煤)的二氧化硅颗粒物(×40, 000)；B：二氧化硅颗粒物局貌(×100, 000)；C：TEM-EDX(C1)。 Silica particles. A: After purification filter membrane (C1 coal) of silicon dioxide particles (×40, 000); B: Bureau of silica particles (×100, 000); C: TEM, EDX (C1).

### 2.5 电子显微镜观察结果

对宣威地区的肺癌患者的术后标本进行电子显微镜观察。结果显示，在宣威市来宾地区的肺癌患者术后病理标本中，可观察到有纳米级细颗粒存在，主要分于患者肺部组织的细胞核内，部分在胞浆内(图 6)。

**6:**
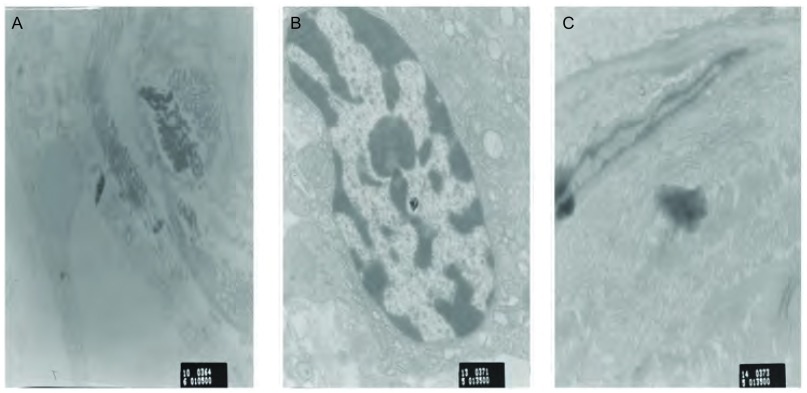
肺内细颗粒物。A：宣威市来宾镇肺癌病理切片电镜图；B：细颗粒物局部全貌；C：细颗粒物形态。 The fine particulate matter of lung. A: Lung cancer electron micrographs of laibin town Xuanwei; B: Partial picture of fine particulate matter; C: Form of fine particulate matter.

## 3 讨论

肺癌已成为制约云南省宣威地区社会经济发展和影响社会民生的重要因素。该地区是中国乃至世界肺癌的高发区，寻找肺癌的发病原因以及发病机制成为研究的热点和难点。本研究结果提示：肺癌高发区出产的C1煤层的燃烧的粉尘较肺癌相对低发的地区的煤燃烧后的可吸入颗粒物浓度高。刘等^[6]^对不同煤进行了燃烧实验发现，不同热力作用，释放的可吸入颗粒物不同。不同燃烧温度下生成的PM2.5质量粒径分布均呈双峰分布；随着燃烧温度的提高，PM2.5质量浓度增加。吕等^[7]^研究发现煤粉越细、燃烧时间越长、燃烧温度越高，生成的PM10、PM2.5、PM1的量均越大；煤粉中添加CaO后，对颗粒物的凝并和团聚起到了一定的作用，降低了可吸入颗粒物的排放量。徐等^[8]^研究表明，CaO对颗粒物凝并和团聚有一定作用，使得静电除尘器前粗颗粒物所占烟尘总量的百分比增加。燃烧室中加入吸附剂能通过形成复杂化合物团聚超细颗粒物，并有效防止某些金属物质气化，并建议对于不同反应器使用几种不同种类的吸附剂(例如硅铝矿石、铝土矿、石灰石和熟石灰)对超细颗粒物进行团聚控制^[9]^。可以对宣威肺癌高发区燃煤中加入CaO等吸附剂来降低燃烧产生的PM10、PM2.5的释放，从而降低人体呼吸道对可吸入颗粒物的吸入暴露。

研究结果提示: C1煤层燃烧产物有氧元素以及硅聚集，三组滤膜上均发现了碳，硫，氧的聚集，此外还发现钙元素、铝元素。在空气采样中，碳元素多为黑炭(black carbon, BC)。BC通常被认为是PM2.5的组分之一^[10]^。Jacobsen等^[11]^对ApoE-/-小鼠气管滴注CB，经彗星实验，发现CB能够诱导支气管肺泡灌洗液中细胞DNA损伤。Totsuka等^[12]^对C57BL/6J小鼠气管滴注CB，经彗星实验发现，CB能够诱导肺脏出现DNA损伤。硫元素则多以化合物形式存在，以硫铝酸钙和硫化钙存在较为多见^[13]^。形成一些颗粒大小不等的颗粒物，对粘膜长生机械损伤的作用。刘等^[14]^研究表明，在煤燃烧过程中，由于可吸入颗粒物结构分子易被破坏，则较易从煤中挥发；以无机态存在的微量金属元素，在较高的温度下才会有较高的挥发性^[15]^。此次发现的Al、Ca元素可能来自无机物高温分解。但此次检测中未见明显的有害重金属物质的检出，由此粗略推测，该地区的肺癌的发生与烟煤中的重金属元素并没有明显的相关性。

研究结果提示：发现C1煤层燃烧后，留在滤膜表面的杂质较多，且形态不规整，被呼吸道吸入后，颗粒物还可造成呼吸道粘膜组织的损伤^[16]^；进一步加重炎症反应，最终可能导致气管内皮癌变^[17]^。而Timblin等^[18]^发现用低浓度PM2.5染毒肺泡上皮细胞后，伴随细胞增生，原癌基因*c-jun*、*junB*、*fra1*和*fra-2*以及凋亡相关基因的表达也升高，这些原癌基因的过度表达将导致细胞增生过度，同时造成细胞恶性转化。颗粒物通过本身的成分，如金属、多环芳烃和碳黑等刺激机体可产生的活性氧化成分，另外机体在清除颗粒物的过程中，也可以产生这种大量的活性氧和活性氮产物，这些活性成分进一步激活了呼吸系统靶细胞的氧化反应信号通路^[19]^。细颗粒物含有的有机物作用于呼吸道上皮细胞，激活信号转导，从而活化癌基因，影响调节基因和抑癌基因的表达，增加癌变发生的危险性^[20]^。在C1煤层的燃烧产物中，找到硅和氧的化合物，对其进行测量后可见纳米级的SiO_2_。既往研究表明，纳米级的SiO_2_有很强的细胞毒性，李等^[5]^研究发现，SiO_2_具有氧化损伤的作用，粉尘中的SiO_2_可能在吸入人体后产生氧化损伤的毒性作用，在宣威地区肺癌发生过程中起到了一定的作用。暴露在纳米SiO_2_中可增加ROS水平和还原型谷胱甘肽水平。增加生产丙二醛和乳酸脱氢酶的释放，从细胞层面显示脂质过氧化和膜损伤^[21]^。

纳米SiO_2_颗粒可致大鼠肺巨噬细胞和肺泡Ⅱ型上皮细胞超微结构改变，随粒径的减少，超微结构改变越明显。纳米SiO_2_颗粒可致大鼠肺组织细胞的氧化应激和炎症反应。纳米SiO_2_颗粒可致肺组织细胞双核及多核发生。可通过Fas/Fast死亡受体通路诱导细胞凋亡^[22]^。孙等^[23]^曾在动物实验中发现大鼠气管及肺组织损伤与大鼠吸入污染物的时间长短及浓度呈正相关性关系。纳米SiO_2_在宣威肺癌的发生发展产生促进作用^[24]^。

在宣威肺癌患者的术后标本中发现纳米级细颗粒物，主要分布于细胞核等处。进一步佐证了细颗粒可能在宣威地区肺癌的发病过程中起到了某种作用。Sørensen等^[25]^研究表明，中等程度的PM的浓度可以诱导氧化性DNA损伤。可以推测细颗粒物是直接所用与细胞核，从而造成细胞的DNA损伤的。细颗粒物可能通过机械损伤、免疫毒性、氧化损伤及其附着的有害化学物质作用，最终导致细胞癌变^[26]^。

综上，在宣威地区中有不同流行病学背景的煤样，进行燃烧试验后，C1煤层燃烧的产物较其他肺癌低发地区杂质更多，颗粒物中存在Si，氧元素的富集，部分的滤膜上发现游离的纳米级SiO_2_。当地肺癌的高发可能与当地人使用烟煤相关。与细颗粒物的机械损伤、氧化活性、附着的有害化学物质、癌基因激活及DNA的损伤相关。我们从改善烟煤燃烧的环境，燃烧温度，添加CaO等催化剂等方面来入手，为宣威地区肺癌高发区的防癌、控癌提供理论依据。
